# Floral Color Change Mediated by Pollen Input–Output and Its Manipulation of Pollinator Foraging Behavior in Heterostylous Plant *Arnebia guttata* (Boraginaceae)

**DOI:** 10.1002/ece3.72599

**Published:** 2025-12-02

**Authors:** Dengfu Ren, Rongqian Zheng, Yaxin Zhai, Aiqin Zhang

**Affiliations:** ^1^ Xinjiang Key Laboratory of Biological Resources and Genetic Engineering College of Life Science and Technology, Xinjiang University Urumqi China; ^2^ Karamay Senior High School Karamay China

**Keywords:** *Arnebia guttata*, ecological function, floral color change, heterostyly, self‐pollen deposition

## Abstract

The phenomenon of floral color change (FCC) in angiosperms is frequently associated with pollination events. However, research on triggers, functions, and ecological effects of FCC still remains confined to phenomenological speculation or model‐based validation due to the overlap between natural senescence and multiple ecological roles, as well as the difficulties in quantitative analysis of pollen transfer. *Arnebia guttata,* a typical distylous species characterized by five gradually fading purple spots on its corolla, serves as an excellent paradigmatic system for studying the triggers and adaptability of FCC, owing to its heteromorphic pollen‐stigma morphology. Therefore, we investigated the flower syndrome, dynamic changes of spots under controlled pollination, pollinator visiting behavior and pollen deposition mediated by artificial spots. The results showed that emasculating and bagging, as well as inter‐ or intra‐morph pollination, can accelerate or delay the time of spots fading. When artificial spots were added to some spotless older flowers to mimic natural ones, these flowers, whether with natural or artificial spots, attracted significantly more pollinators than those without spots. Additionally, the number of incompatible pollen grains on the stigmas of both L‐morph and S‐morph flowers increased significantly, while the compatible pollen grain counts remained unchanged. In conclusion, as a typical distylous plant with weak self‐incompatibility in the S‐morph, FCC in *A. guttata*, which is influenced by pollen input–output dynamics, plays a critical role in reducing self‐pollen deposition. For the first time, the study systematically evaluated the induction and ecological function of FCC through employing the manual control experiment, quantitative analysis of stigma pollen deposition, and investigation of pollinator visitation behavior in a distylous population.

## Graphical abstract

The inducement, functions of flower color change (FCC) and its ecological effects have been studied by controlling pollination, accurate statistics of pollen transfer and visiting behavior in a typical distylous species. The results showed that limiting pollen input–output can delay fading time of spots, adding fake spots to old flowers can improve visiting frequency, but increase stigma self‐ pollen deposition. FCC which was influenced by pollen input–output dynamics plays a critical role in reducing self‐pollen deposition.

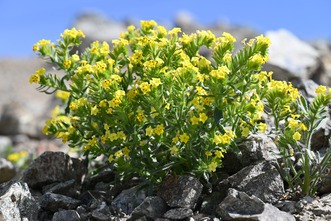

## 1 Introduction

Floral color serves as a visual signal from plants to pollinators, exhibiting high diversity through interspecific differences, intraspecific variations in floral pigmentation or color changes in specific floral structures (Gori 1989; Hodges et al. 2003; Tang and Huang 2012; Kameoka et al. 2016; Zhou et al. 2023). Floral color change (FCC), a common floral display strategy characterized by chromatic shifts during anthesis, typically occurs when aging flowers with diminished rewards remain on the plant rather than being abscised (Weiss 1995). This phenomenon is widespread in flowering plants, and has been documented in at least 78 plant families, such as Cruciferae, Papilionaceae and Boraginaceae (Weiss and Lamont 1997; Zhang et al. 2017). FCC is often influenced by multiple factors, including pollinator selection, natural aging or environmental conditions, and playing essential ecological functions (Weiss 1995; Oberrath and Böhning‐Gaese 1999; Tang and Huang 2010; Vaidya et al. 2018; Humphreys and Skema 2023). For instance, in *Davidia involucrata*, bracts change from green to white to enhance pollinator attraction under certain conditions (Sun et al. 2008). In other cases, FCC is believed to function in reducing excessive visitation to senescing flowers, and improving pollinators' foraging efficiency, because it tends to maintain the flowers' long‐distance visibility while reducing their short‐range attractiveness to insects (Ollerton et al. 2007; Ohashi et al. 2015). The coexistence of two distinct floral colors within an individual plant is regarded as a flower display strategy, a signaling system refined by pollinator behavior (Kudo et al. 2007; Makino and Ohashi 2016).

However, alternative viewpoints have also been proposed, suggesting that FCC is a normal programmed aging process unrelated to pollinator selection, as many plant organs undergo color changes during senescence, such as leaves and fruits (Casper and LaPine 1984; Lamont and Collins 1988). Some researchers even argue that intraspecific floral color variation perceived by humans may be invisible to pollinators, thereby escaping pollinator‐mediated direct selection (Paine et al. 2019). Furthermore, FCC is also related to environmental heterogeneity and flower gender transformation (Vaidya et al. 2018; Humphreys and Skema 2023). For instance, in the aquatic plant *Butomus umbellatus* (Butomaceae), FCC from red to pink occurs in response to water level fluctuations, which is not linked to pollinators' foraging behavior (Tang and Huang 2010). Similarly, Jabbari et al. (2013) found that FCC in the protandrous species *Saponaria officinalis* correlates with both gender transition and ambient light conditions. Although there was no difference in the number of visitors to flowers of different colors or genders, a notable preference for pink female‐stage flowers was observed (Jabbari et al. 2013). Therefore, three primary hypotheses have been proposed to explain the causes of FCC: the pollinator‐induced hypothesis (Sugahara et al. 2010; Pereira et al. 2011), the time‐dependent hypothesis (Casper and LaPine 1984; Lamont and Collins 1988) and the environmental heterogeneity hypothesis (Tang and Huang 2010; Anderson et al. 2013). The existence of these competing hypotheses indicates that the mechanisms driving FCC are multifaceted. Therefore, the ecological significance of floral color variation across different plant groups and the underlying driving factors still require extensive research (Paine et al. 2019).

Analyzing pollinator's visiting behavior and the resulting ecological effects is an important means for evaluating the ecological function of FCC. Currently, three hypotheses have been proposed from distinct perspectives to explain the adaptive consequences of FCC: improving pollination efficiency, reducing geitonogamy and avoiding reproductive interference. For example, Weiss (1991, 1995) compared the visiting behavior of pollinators in several species possessing FCC and found that the visiting frequency of pollinators declined after flowers changed color. In *Pedicularis monbeigiana* (Orobanchaceae), the proportion of purple (old) flowers within inflorescences showed a significant negative correlation with pollinator visitation frequency. These findings suggest that FCC may serve as an adaptive mechanism to enhance inter‐individual pollen transfer and reduce the pollen deposition from the same plant by diminishing the attraction of older flowers (Sun et al. 2005). However, other studies contend, that FCC did not improve pollination efficiency or decrease geitonogamy. Instead, its primary function may lie in preventing pollinators from revisiting flowers visited through FCC, thereby protecting post‐pollination reproductive processes like pollen tube growth and embryo formation. For example, in *Fuchsia excorticata* (Onagraceae), flowers persisted after changing color to red because pollen tube growth requires at least 3 days to reach the ovary, while corolla tube and style shedding takes at least 1.5 days (Delph and Lively 1989). The effective pollen transfer among individuals largely depends on pollinators and plants' regulation of pollinator foraging behavior. The triggers, functions, and ecological effects of FCC reflect the interaction between plants and pollinators. FCC, which aims to reduce excessive visits to old flowers and improve pollination efficiency, may increase cross‐pollen transfer while reducing self‐pollen deposition on stigmas. However, due to the simultaneous occurrence of pollen input–output, as well as the difficulty in identifying cross‐ and self‐pollen on stigmas, it is difficult to conduct quantitative analysis of pollen transfer in populations with single pollen‐stigma morphology.

*Arnebia guttata is* a distylous plant that is distributed in arid deserts characterized by five dark purple spots on the corolla of per newly opened flowers, which fade away in the afternoon of the same day. It remains unclear whether the FCC is linked to pollination events, and what ecological significance it holds. Due to the dimorphism of pollen size, *A. guttata* provides an ideal model for studying the triggers and ecological effects of FCC, and verifying relevant hypotheses: pollinator‐mediated selection and avoidance of geitonogamous pollination through quantitative analysis of pollen transfer. Therefore, we conducted field studies in a natural population of *A. guttata* by investigating its flowering habits, the dynamics of FCC mediated by pollen input–output, pollinators' foraging behavior and pollen deposition on stigmas following artificial manipulation of floral spots (e.g., adding artificial spots to old flowers without spots), to address the following questions: (1) Is FCC related to pollination events such as the deposition of pollen on stigmas and pollen dispersal? (2) Does FCC alter pollinator visiting behavior? (3) How does FCC affect pollen deposition, whether it improves pollination efficiency or reduces geitonogamy? The research on the above questions will provide a new perspective for an in‐depth understanding of the interaction between plants and pollinators, the inducement and ecological significance of FCC.

## 2 Materials and Methods

### 2.1 Study Site and Study Species

**FIGURE 1:**
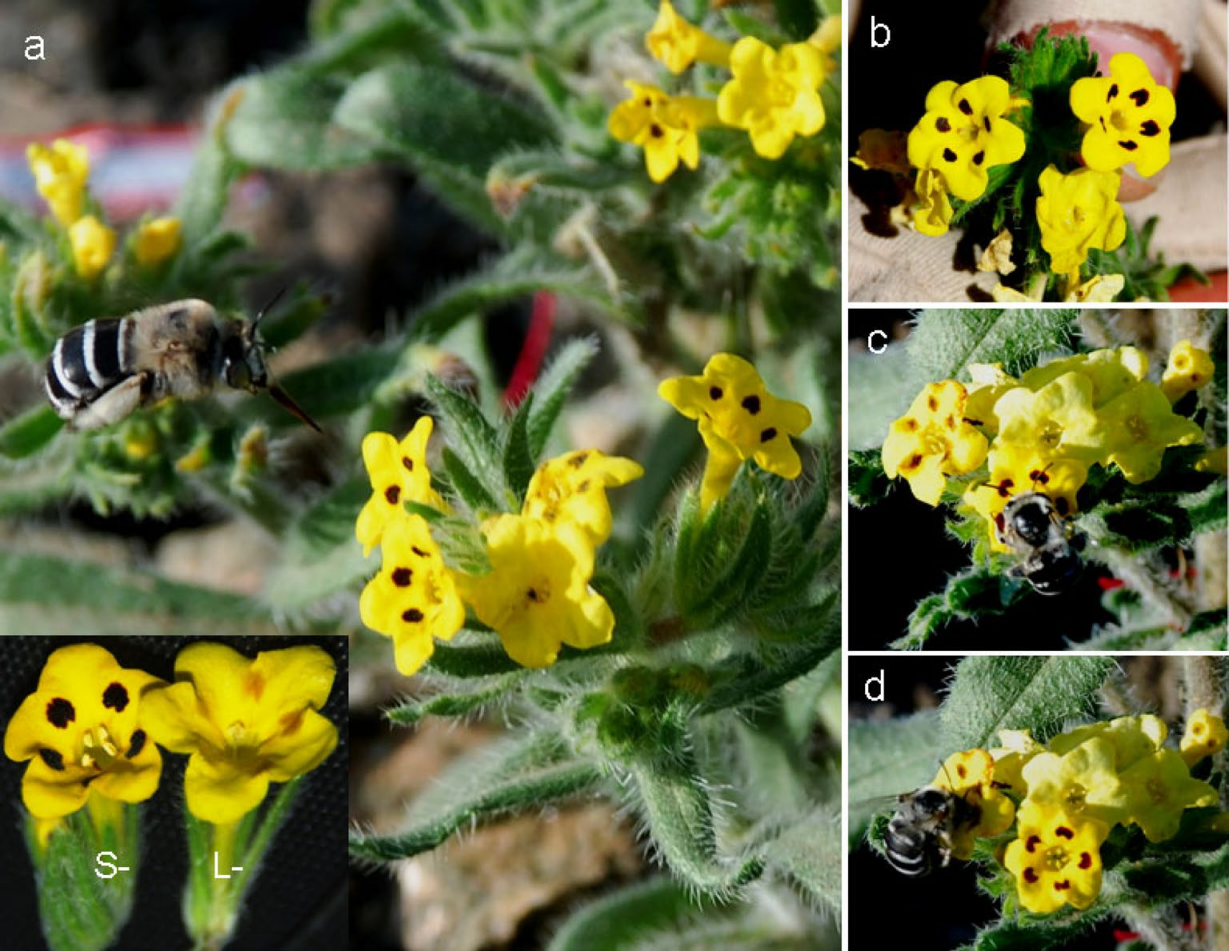
The floral morphs, flower colors and pollinator of *Arnebia guttata* . (a) The main pollinator *Amegilla* sp. and two floral morphs (L‐ and S‐ morph) of *A. guttata* . (b) Two newly opened flowers with five dark purple spots and a spotless old flower. (c) A pollinator that is visiting a flower with natural spots; (d) A pollinator that is visiting a flower with artificial spots.

*Arnebia guttata* Bge. (Boraginaceae), a perennial herb, grows mainly in deserts, gravel slopes or Gobi in China, Northwest India, Pakistan, Afghanistan and Siberia (http://www.iplant.cn/info/). The plant has many branches and is covered with long, thick bristles and short hairs. It blooms from June to August and produces 3–10 cm long falciform cymes composed of dozens of yellow tubular flowers. A singular flower can last for 2 days, with five obvious purple spots near the throat of the flower tube, in which the purple spots will fade away in the afternoon of the same day, while the flower will close in the afternoon of the next day (Figure 1). As a distylous species, its populations comprise two distinct floral morphs of long‐styled (L‐morph) and short‐styled morphs (S‐morph) characterized by a reciprocal arrangement of anthers and stigmas, which exhibit distinct pollen‐stigma morphological features. This study was conducted in a wild population with thousands of individuals in Qinghe County, east of Altay Mountain (46°18′40″N, 90°51′7″E, 1400 m asl.), in Xinjiang, China.

### 2.2 Investigation of Floral Morph Ratio and Floral Traits

In the flowering period of *A. guttata* , 10 sample plots of 6 m^2^ each were randomly set within the study population, and all individuals were examined by counting the number of S‐morph and L‐morph individuals within each plot. Meanwhile, 20 flowers of each floral morph were randomly harvested from different individuals and measured for corolla open diameter, corolla tube diameter, corolla tube length, pistil height and stamen height using digital calipers with an accuracy of 0.02 mm.

### 2.3 Detection of Heteromorphic Incompatible System

To assess heteromorphic incompatibility, 30 individuals of each floral morph were randomly selected in the fully blooming period, and 5–6 unopened flowers were randomly labeled for each individual and subjected to the following pollination treatments respectively: (1) apomixis (emasculation and bagging); (2) artificial self‐pollination and bagging; (3) intra‐morph pollination and bagging (L × L, S × S); (4) intermorph pollination and bagging (L × S, S × L); and (5) control (open pollination). We assessed fruit sets in the various pollination treatments after fruit ripening.

### 2.4 FCC Mediated by Pollen Input/Output

To investigate the relationship between FCC and pollen input/output, 20 individuals of each floral morph were randomly selected, and 4 unopened flowers were labeled for each individual and conducted the following pollination treatments respectively: (1) emasculation and bagging (pollen removal only); (2) bagging only (no pollen receipt and removal); (3) artificial intermorph‐pollination and bagging (pollen receipt only); (4) open pollination as control (both pollen receipt and removal). The fading status of spots and the time of spots fading for each pollination treatment were recorded every 2 h in the early stage and every 30 min in the later stage from 9:00 AM to 21:00 PM of the first day, and continued this observation until the next day.

### 2.5 Examination of the Advertising and Ecological Effects of FCC

To examine the advertising and ecological effects of FCC, the visiting behavior and stigmatic pollen deposition were investigated in a manipulated plot (where artificial spots were applied to partial spotless old flowers to mimic the natural spots using a purple water‐soluble marker with water as solvent without any odor) and a natural plot (control). These two sample areas had the same size and equal ratios of flower types, both of which were of equal size (about 10 m^2^) and ratios of L‐/S‐morphs. To maintain the same ratio of flowers with natural, spotless and artificial spots on each individual, we removed excess flowers with tweezers. Data were recorded on the floral morph (L‐morph and S‐morph), the floral color (with natural spots, spotless and artificial spots), and the number of flowers observed and visited. The visiting frequencies for flowers with different floral morphs and colors were calculated. Meanwhile, the number of compatible and incompatible pollen on stigmas (distinguished by pollen size and shape) was counted after 4–5 h of visiting in two plots. These observations were repeated for three consecutive days in the sunny windless morning.

### 2.6 Statistical Analysis

The floral morph ratio was compared using a Chi‐square test to examine the existence of deviations from isoplethy (1:1). Floral traits (flower size parameters) were compared between floral morphs using a generalized linear model (GLM) with a Gaussian distribution and identity link function. To examine the mating system of *A. guttata* , a GLM with a binomial distribution and logit link function was used to compare the fruit sets among pollination treatments. To examine the effects of pollen input–output on FCC, the time of spots fading was compared among pollination treatments using a GLM with a Gaussian distribution and identity link function. To examine the effects of floral colors (with natural spots, spotless and artificial spots) on pollinators' preference, we used a GLM with a Gaussian distribution and identity link function for comparing visiting frequency, with floral morphs (L‐morph and S‐morph), floral colors (with natural spots and spotless), treatments (natural plot and manipulated plot) and their interaction as fixed factors. In this model, only natural spotted and spotless flowers were considered regardless of artificial spotted flowers because of the numeric problem when making a comparison between natural (floral color with 2 levels of natural spots and spotless) and manipulated plots (floral color with 3 levels of natural spots, spotless and artificial spots). The effects of floral color on compatible, incompatible and total pollen deposition on stigmas were examined using the GLM with a Poisson distribution and log link function. The effects of floral color on the proportion of compatible pollen on stigmas were analyzed using a GLM with a binomial distribution and logit link function. All analyses were performed using SPSS 20.0. Data are presented as mean ± SE.

## 3 Results

### 3.1 Floral Morph Ratio and Floral Traits

The population was composed of L‐ and S‐morphs exhibiting reciprocal arrangements of stigma and anther height. A total of 160L‐ and 242S‐morphs were recorded in the surveyed quadrats. The relative abundance of S‐morphs was significantly higher than that of the L‐morph (Wald *χ* ^2^ = 16.726, *p* < 0.001). *Arnebia guttata* was identified as a typical distylous species with strict reciprocal herkogamy. This was characterized by the significant differences in pistil and stamen height between floral morphs, and the matched heights between stigma in L‐morph and anther in S‐morph (higher sex organs), as well as anther in L‐morph and stigma in S‐morph (lower sex organs). Furthermore, no inter‐morph differences were detected in corolla open diameter, corolla tube diameter and corolla tube length (Table 1).

**TABLE 1:** The floral characteristics of *Arnebia guttata* (Mean ± SD mm, *N* = 20).

	Corolla open diameter	Corolla tube diameter	Corolla tube length	Pistil height	Stamen height	*χ*2 and *p* value of sex organs height	
						Higher	Lower
L‐morph	10.13 ± 0.15^a^	1.60 ± 0.02^a^	12.00 ± 0.18^a^	12.62 ± 0.2^a^	7.89 ± 0.12^b^		
S‐morph	10.50 ± 0.26^a^	1.63 ± 0.04^a^	12.34 ± 0.25^a^	7.69 ± 0.14^b^	12.30 ± 0.22^a^		
*χ* ^2^	1.458	0.486	1.280	421.790	316.868	1.189	1.228
*p*	0.227	0.486	0.258	0.000	0.000	0.276	0.268

*Note:* Different lowercase letters indicate a significant difference at the 0.05 level in the same column.

### 3.2 Heteromorphic Incompatibility System

*Arnebia guttata* species possesses a heteromorphic incompatibility system, in which there were significant differences among different pollination treatments (Wald *χ* ^2^ = 40.327, *p* < 0.001). It was manifested that the flowers produced no or a few fruits after emasculation and bagging, as well as intra‐morphic and artificial self‐pollination, exhibited no apomixis, self, or intra‐morphic compatibility in L‐morphs, and weak self‐ and intra‐incompatibility in S‐morphs. The intermorph‐pollination was compatible (L × S: 92.11% ± 4.43%; S × L: 97.44% ± 2.56%; *p* = 0.702), with a high fruit set. There was no pollination limitation within the observed population due to the absence of significant differences between inter‐morphic pollination and open pollination (*p* = 0.117). In all pollination treatments the fruit sets were not affected by the floral morph (Wald *χ* ^2^ = 0.000, *p* = 0.999) and the interaction between floral morph and pollination treatment (Wald *χ* ^2^ = 0.153, *p* = 0.997), but were influenced by pollination treatment (Wald *χ* ^2^ = 40.327, *p* < 0.001) (Table 2).

**TABLE 2:** Fruit sets under different pollination treatments in *Arnebia guttata* (mean ± SE).

Treatments	L‐morph		S‐morph	
	Sample size (flower)	Fruit set (%)	Sample size (flower)	Fruit set (%)
Emasculated and bagged	30	0.00^c^	29	0.00^c^
Artificial self‐pollination	28	3.57 ± 3.57^cd^	26	15.38 ± 7.22^bd^
Intra‐morph pollination	29	0.00^c^	37	18.92 ± 6.53^b^
Inter‐morph pollination	38	92.11 ± 4.43^a^	39	97.44 ± 2.56^a^
Open pollination	31	90.32 ± 5.40^a^	22	90.91 ± 6.27^a^

*Note:* Different lowercase letters indicate a significant difference at the 0.05 level.

### 3.3 FCC Mediated by Pollen Input/Output

Under open pollination conditions, all newly opened flowers exhibited dark purple spots on their corollas, in which the spots would gradually fade away and disappear at about 17:00–18:00 PM on the same day of flowering. Following these spots' disappearance, the flowers continued to bloom till 17:00–18:00 PM of the next day. No significant differences were observed in the time of spots fading between the two floral morphs in *A. guttata* . However, there were significant differences under controlled pollination (Wald *χ* ^2^ = 160.154, *p* < 0.001). When the flowers were emasculated only (pollen removal only) or pollinated artificially using inter‐morph pollen without emasculation (pollen receipt only), the fading time of the spots was significantly longer than that of open (natural) pollination. In both floral morphs, the corolla spots disappeared at about 20:00 on the same day (pollen removal only: L‐morph: *p* = 0.025; S‐morph: *p* = 0.018; pollen receipt only: L‐morph: *p* = 0.006; S‐morph: *p* = 0.011). When bagged solely for pollen isolation (without pollen receipt and removal), the corolla spots of both floral morphs faded around 17:00 in the following day, which was the slowest among pollination treatments (all *p* < 0.001). And no significant difference in fading time was detected between the L‐ and S‐morphs (*p* = 0.556). These results demonstrated that changes in corolla spots were associated with stigmatic pollen receipt and anther pollen dispersal. Statistical analysis revealed that the fading time was not influenced by floral morph (Wald *χ* ^2^ = 0.054, *p* = 0.816) nor by the interaction between floral morph and pollination treatment (Wald *χ* ^2^ = 0.311, *p* = 0.958), but was significantly affected by the pollination treatment (Wald *χ* ^2^ = 160.154, *p* < 0.001).

### 3.4 The Advertising and Ecological Effects of FCC

#### 3.4.1 Visiting Frequency Mediated by Artificial Spots

**FIGURE 2:**
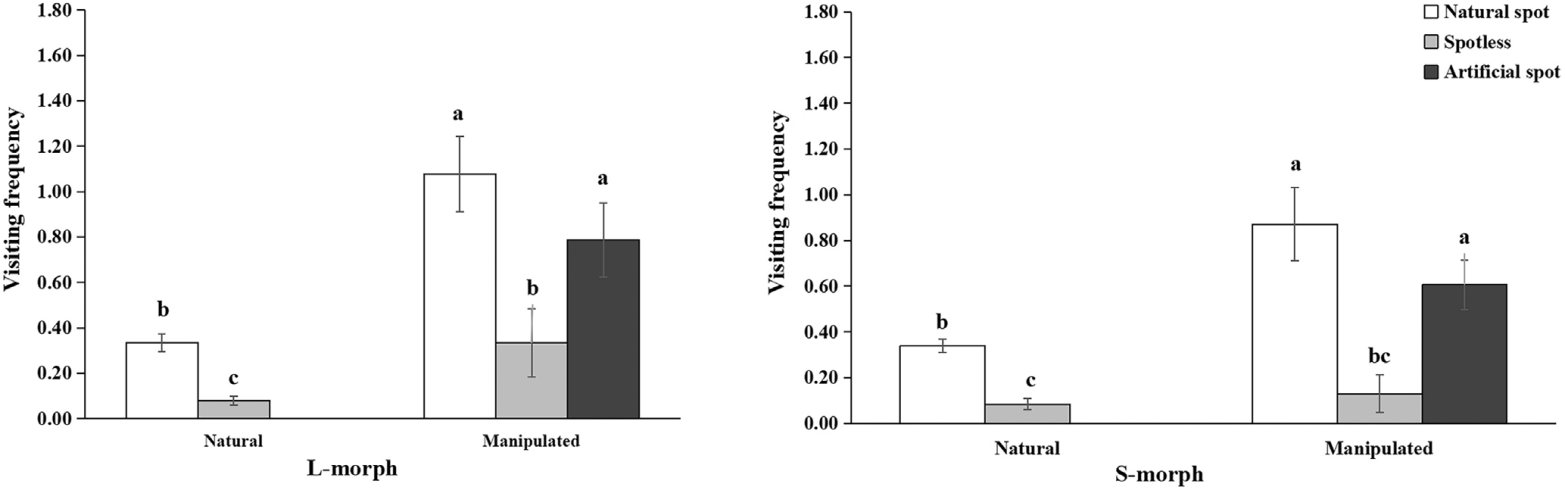
The visiting frequency of different floral colors (with natural spots, spotless and artificial spots) and floral morphs under natural and manipulated plots in *Arnebia guttata* . Different lowercase letters indicate a significant difference at the 0.05 level among floral colors within plot or between plots by a multiple comparison under GLM.

In natural populations, based on a 38‐h observation, we identified a species of *Amegilla* as the primary pollinator of *A. guttata* , with butterfly and hawkmoth serving as occasional visitors. All observed pollinators were long‐tongued insects and could efficiently facilitate intermorph pollination. Under natural pollination conditions, observations of 38 L‐morph and 41 S‐morph individuals revealed that the flowers with dark purple spots, regardless of L‐ or S‐ morphs, were visited more frequently than spotless old flowers (L‐morph: *p* = 0.008; S‐morph: *p* = 0.030) (Figure 2).

When adding artificial spots to spotless old flowers, the visiting frequencies of manipulated plots significantly increased compared to the natural plot (Waldχ2 = 23.984, *p* < 0.001), no matter in L‐morph or S‐morph (L: *p* = 0.026; S: *p* = 0.029) (Figure 2). The visiting frequencies were significantly affected by floral color (with natural spots and spotless) and treatment (natural and manipulated plots), but not by floral morph. A significant interaction was observed between floral color and treatment on visiting frequencies. In contrast, no significant interactions were found between floral color and floral morph or between floral morph and treatment on visiting frequencies (Table 3).

**TABLE 3:** Comparison and analysis of visiting frequency between natural and manipulated plots (excluding artificial spotted flower in manipulated plot).

Source of variation	df	Wald *χ* ^2^	*p*
Visiting frequency			
Intercept	1	163.54	0.000
Floral morph (L‐ and S‐morph)	1	2.480	0.115
Floral color (flower with natural spots and spotless)	1	61.886	0.000
Floral morph × Floral color	1	0.000	0.983
Treatment (natural and manipulated plots)	1	38.602	0.000
Floral morph × Treatment	1	2.703	0.100
Floral color × Treatment	1	14.835	0.000
Floral morph × Floral color × Treatment	1	0.000	0.994

In the manipulated plots, visiting frequencies did not differ significantly between flowers with natural spots and artificial spots in either the L‐morph (*p* = 0.071) or the S‐morph (*p* = 0.201) (Figure 2). The visiting frequencies were influenced significantly by the floral color (with natural spots, artificial spots and spotless), but not by floral morph, and the interaction between floral morph and floral color (Table 4). The results indicated these spots on the corolla showed an obvious advertising effect on the pollinator visiting behavior.

**TABLE 4:** Comparison of visiting frequency in manipulated plots (adding artificial spots).

Source of variation	df	Wald *χ* ^2^	*p*
Visiting frequency			
Intercept	1	109.152	0.000
Floral morph (L‐ and S‐morph)	1	2.625	0.105
Floral color (flower with natural spots, artificial spots and spotless)	2	23.984	0.000
Floral morph × Floral color	2	0.008	0.996

#### 3.4.2 Pollen Deposition on Stigmas Mediated by Artificial Spots

**FIGURE 3:**
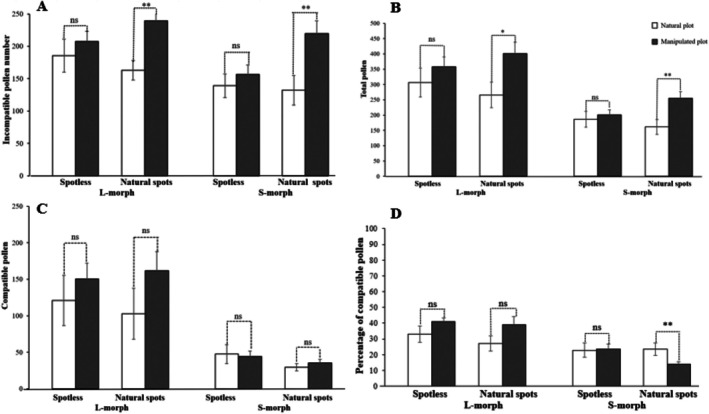
The number of incompatible, total pollen grains, compatible pollen deposited and the percentage of compatible pollen on stigmas of different floral colors (with natural spots, artificial spots, and spotless) of L‐ and S‐morph in *Arnebia guttata* between natural and manipulated plots. Bars with asterisks indicate a significant difference between variables (*p < 0.05; **p < 0.01; ns, *p* > 0.05) according to generalized linear models (GLM).

After adding artificial spots to spotless old flowers, the stigma pollen loads showed significant differences between natural and manipulated plots (Wald *χ* ^2^ = 46.716, *p* < 0.001). Specifically, the incompatible (L‐morph: *p* = 0.010; S‐morph: *p* = 0.010) and total pollen number on stigmas (L‐morph: *p* = 0.037; S‐morph: *p* = 0.010) increased significantly in L‐ and S‐ morph with natural spots (Figure 3A,B). However, the number of compatible pollen grains did not change in L‐ and S‐morph (L‐morph: *p* = 0.254; S‐morph: *p* = 0.482) (Figure 3C). The percentage of compatible pollen showed no significant change in L‐morph (*p* = 0.126) but decreased markedly in S‐morph (*p* = 0.010) (Figure 3D). The number of compatible pollen grains and its percentage on stigmas were influenced solely by floral morph, However, both the incompatible pollen and total pollen number were affected by floral morph and treatment (Table 5).

**TABLE 5:** Comparison of pollen deposition between natural and manipulated plots (excluding artificial spotted flowers in manipulated plot).

Source of variation	df	Wald χ^2^	*p*
Incompatible pollen			
Intercept	1	13131.137	0.000
Floral morph (L‐ and S‐morph)	1	5.741	0.017
Floral color (flower with natural spots and spotless)	1	0.691	0.406
Floral morph × Floral color	1	0.578	0.447
Treatment (natural and manipulated plots)	1	9.666	0.002
Floral morph × Treatment	1	0.142	0.706
Floral color × Treatment	1	3.359	0.067
Floral morph × Floral color × Treatment	1	0.104	0.748
Compatible pollen			
Intercept	1	3038.576	0.000
Floral morph	1	63.803	0.000
Floral color	1	1.679	0.195
Floral morph × Floral color	1	1.006	0.316
Treatment	1	1.596	0.207
Floral morph × Treatment	1	0.815	0.367
Floral color × Treatment	1	0.632	0.426
Floral morph × Floral color × Treatment	1	0.001	0.977
Total pollen			
Intercept	1	16,267.626	0.000
Floral morph	1	33.993	0.000
Floral color	1	0.035	0.852
Floral morph ×Floral color	1	0.125	0.723
Treatment	1	9.957	0.002
Floral morph × Treatment	1	0.009	0.926
Floral color × Treatment	1	3.392	0.066
Floral morph × Floral color × Treatment	1	0.130	0.718
Percentage of compatible pollen			
Intercept	1	154.186	0.000
Floral morph	1	46.316	0.000
Floral color	1	3.122	0.077
Floral morph × Floral color	1	1.980	0.159
Treatment	1	0.261	0.609
Floral morph × Treatment	1	1.267	0.260
Floral color × Treatment	1	0.063	0.802
Floral morph × Floral color × Treatment	1	0.016	0.900

**FIGURE 4:**
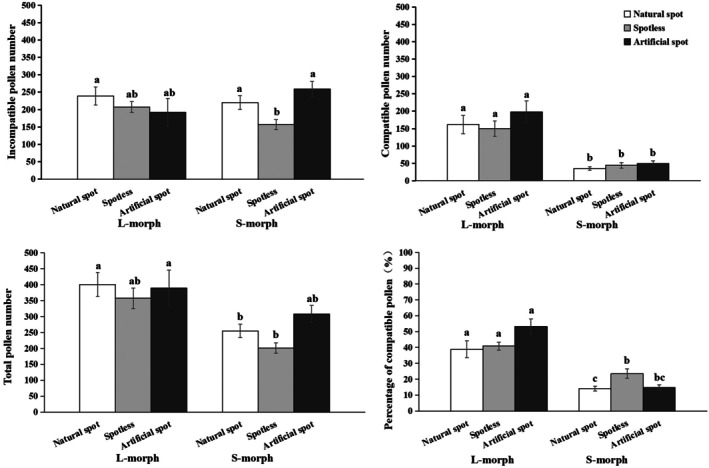
The number of incompatible, compatible, total pollen grains and the percentage of compatible pollen on stigma in different flower colors of L‐ and S‐morph of *Arnebia guttata* under manipulated plot. Different lowercase letters indicate a significant difference at the 0.05 level within or between floral morphs.

Meanwhile, we also compared the pollen deposition data of different floral colors (with natural spots, artificial spots and spotless) in manipulated plots (after adding artificial spots). There was no difference in the number of incompatible pollen grains (L‐morph: *p* = 0.356; S‐morph: *p* = 0.198), compatible pollen grains (L‐morph: *p* = 0.400; S‐morph: *p* = 0.129), and percentage of compatible pollen grains (L‐morph: *p* = 0.059; S‐morph: *p* = 0.746) between artificial and natural spotted flowers (Figure 4). The number of incompatible pollen grains was not affected by the floral morph, floral color and the interaction between floral morph and floral color, while that of compatible pollen grains and total pollen grains was significantly affected by floral morph, and the percentage of compatible pollen grains was affected not only by floral morph, but also by the interaction between floral morph and floral color (Table 6).

**TABLE 6:** Comparison of pollen deposition in manipulated plot (adding artificial spots).

Source of variation	df	Wald *χ* ^2^	*p*
Incompatible pollen			
Intercept	1	9884.965	0.000
Floral morph (L‐ and S‐morph)	1	0.041	0.840
Floral color (flower with natural spots, artificial spots and spotless)	2	3.678	0.159
Floral morph × Floral color	2	5.085	0.079
Compatible pollen			
Intercept	1	5744.853	0.000
Floral morph	1	138.761	0.000
Floral color	2	4.274	0.118
Floral morph × Floral color	2	0.978	0.613
Total pollen			
Intercept	1	16,370.725	0.000
Floral morph	1	22.100	0.000
Floral color	2	5.596	0.061
Floral morph × Floral color	2	2.624	0.269
Percentage of compatible pollen			
Intercept	1	213.952	0.000
Floral morph	1	117.215	0.000
Floral color	2	5.060	0.080
Floral morph × Floral color	2	6.017	0.049

## 4 Discussion

FCC, a dynamic floral display strategy, is often related to pollination events, such as the interaction of plant‐pollinator (Oberrath and Böhning‐Gaese 1999; Sugahara et al. 2010; Tang and Huang 2010; Ohashi et al. 2015). Due to the simultaneous occurrence of multiple factors and the difficulty in identifying the pollen source on stigmas, our understanding of the ecological significance of FCC still relies largely on hypotheses and model‐based validation. In this study, we investigated the inducement and ecological effects of FCC in *A. guttata* , a classic distylous species. Under natural pollination conditions, the newly opened flowers can last for 2 days, with the 5 purple natural spots fading away in the afternoon of the same day, and flowers closed in the afternoon of the next day (Figure 1). The flowers with natural spots received a higher visiting frequency, whereas they had an extremely low visiting frequency after the spots faded away. When the natural pollination process is changed, such as emasculation and bagged, artificial inter‐ or intra‐morph pollination, the original fading time for the spots would be shortened or prolonged. It indicated that the FCC is related to the pollination events and influenced by flower age. When artificial spots were added to some spotless old flowers, the spotted flowers, regardless of natural or artificial spots, exhibited significantly higher pollinator attraction in manipulated plots compared to natural plots. However, the number of incompatible pollen grains on the stigmas of both L‐ and S‐morphs increased significantly, while the number of compatible pollen grains did not change. As a typical distylous species with weak intra‐incompatibility in S‐morph, the FCC aimed at reducing self‐pollen deposition is particularly important.

### 4.1 FCC Mediated by Pollinator and Time‐Dependent Selection

Floral syndrome is closely related to pollinator behavior and the resulting reproductive fitness. However, the understanding of how pollinator selection shapes floral syndromes and how these syndromes, in turn, influence pollinators' behavior remains limited, owing to the fact that many different combinations of flower traits correspond to the same pollination pattern (Barrett 2003; Zhang 2004; Janes et al. 2024). In *A. guttata* , the natural fading time of the spots was between 17:00 and 18:00 on the same afternoon, demonstrating a clear time‐dependent effect. When artificial unilateral receipt (only keeping the realization of female fitness) or removal of pollen (only keeping the realization of male fitness) was performed to replace natural pollen transfer, the fading time of spots was delayed until around 20:00 on the same day. When the receipt and removal of pollen were limited by bagging, the fading time was delayed until 17:00 on the next day. The phenomenon demonstrated that the change of spots on petals was associated with the receipt and removal of pollen, while also being influenced by natural aging processes. In the existing studies, such as *Lotus* *scoparius* (Fabaceae), *Alkanna orientalis* (Boraginaceae), *Euphrasia dyeri* (Orobanchaceae), a relationship between FCC and pollination events, as well as flower age, was also shown (Jones and Cruzan 1999; Nuttman et al. 2006; McGimpsey and Lord 2015). However, these studies put more emphasis on the relationship between female fitness and FCC, and paid less attention to the male fitness of flowers. Our research suggested that FCC is related not only to the achievement of female fitness (pollen receipt), but also to that of male fitness (pollen removal).

The pollinator‐mediated selection hypothesis is often used to explain the change of floral color (Sugahara et al. 2010; Pereira et al. 2011), although the time‐dependent hypothesis and environmental heterogeneity hypothesis have also been supported (Casper and LaPine 1984; Lamont and Collins 1988; Tang and Huang 2010; Anderson et al. 2013). In fact, the pollinator selection hypothesis is often combined with the time‐dependent hypothesis to explain the phenomenon of FCC in many cases (Nuttman et al. 2006; McGimpsey and Lord 2015). For example, the FCC of *Lotus scoparius* (Fabaceae) from yellow to orange can be induced rapidly by a pollination event, but is much slower when no pollination occurs (Jones and Cruzan 1999). In *A. guttata* , the fading time of the spots has obviously been extended after preventing the receipt and removal of pollen through bagging. These phenomena also reflect the combined effect of the two selection forces. This synergistic interaction facilitated the optimal resource allocation and enhanced both female and male fitness.

A close mutualistic interaction exists between plants and their pollinators (Mu et al. 2011; Sun et al. 2018). Specialized pollinators, along with their distinct foraging behaviors and preferences, can act as selective forces driving the evolution of floral syndrome (Whittall and Hodges 2007). Conversely, floral syndromes, including floral color, shape, smell, nectar and pollen rewards, can effectively attract or restrict pollinators visiting at different flowering stages (Jones and Cruzan 1999; Ollerton et al. 2007; Zhang et al. 2017; Wester et al. 2020). In *A. guttata* , the FCC can significantly alter the visiting behavior of pollinators, in which the flowers without spots no longer attract pollinators, with a lower visiting frequency compared with flowers with natural or artificial spots. Like other plants with FCC (Eckhart et al. 2006), the presence or absence of spots can guide pollinators to identify the flowers that offer rewards (with pollen or nectar) and those that do not (without pollen or nectar). The preference of pollinators leads to a difference in visiting frequency and reproductive fitness of individuals with different floral colors, thus mediating the differentiation of floral color (Faegri and van der Pijl 1979; Fenster et al. 2004). Therefore, pollinator‐mediated selection is often the dominant factor in the variation or evolution of floral traits (Moller and Eriksson 1995; Gong and Huang 2009). Our study provides evidence for this hypothesis based on the investigation of the relationship between FCC dynamics and pollen input–output in a species with heteromorphic pollen‐stigma morphology. However, the selection of pollinators is not the sole driver of color polymorphism (Tang and Huang 2010; Vaidya et al. 2018; Humphreys and Skema 2023), and other biotic or abiotic factors can also induce FCC (Mu et al. 2011; Zhang et al. 2017; Paine et al. 2019; Wang et al. 2023). For instance, even self‐pollinating species also exhibit diverse FCC, which can be influenced by environmental conditions, herbivores, soil pH and so on (Vaidya et al. 2018). Therefore, more extensive research across diverse plant species is needed in the future.

### 4.2 The Ecological Effects of FCC

To investigate the ecological effects of FCC, we added artificial spots (false spots) to a subset of spotless old flowers, and found that these flowers achieved a visiting frequency comparable to that of flowers with natural spots. Interestingly, although pollinator visiting frequency increased, this did not lead to more compatible pollen on stigmas. Instead, it resulted in a significant increase in incompatible pollen deposition. This finding suggested that the addition of artificial spots only increased the excessive visiting of pollinators, but couldn't increase the pollination efficiency. Based on these phenomena, we can infer that FCC in *A. guttata* , which was shaped by pollinator selection, can obviously reduce the degree of geitonogamy by limiting the excessive visiting within individuals. Currently, three hypotheses have been proposed to explain the ecological effects of FCC, including improving pollination efficiency, reducing geitonogamy, and avoiding reproductive interference (Ida and Kudo 2003, 2010; Zhang et al. 2012; Makino and Ohashi 2016). Our findings in *A. guttata* have provided empirical support for the hypothesis of reducing geitonogamy. The excessive self‐pollen deposition will occupy the limited space of stigma, interfering with the adhesion and germination of compatible pollen on stigmas in self‐incompatible plants, while leading to inbreeding depression in self‐ compatible plants. Therefore, many reproductive strategies in angiosperms, including herkogamy, heterostyly, dioecism and FCC, are related to reducing the reproductive interference of self‐pollen.

Pollinators' foraging behavior inevitably leads to geitonogamy. When visiting frequency is moderate, pollinators can dilute the proportion of self‐pollen on insects' body surfaces by visiting among individuals. However, when visiting excessively, pollinators' body surfaces will carry a large amount of self‐pollen from the same individual, resulting in excessive self‐pollen deposition. Therefore, excessive visiting is not a beneficial service, but rather a form of reproductive interference. It directly decreases the female and male fitness of individuals by intensifying self‐pollen deposition, causing stigmatic physical damage, and leading to pollen discounts. Meanwhile, it will also reduce the foraging efficiency of the pollinators. Therefore, plants often regulate the visiting behavior of pollinators through visual signals of FCC, stopping the provision of floral rewards, and even closing the petals to prevent excessive visiting (Faegri and van der Pijl 1979; Fenster et al. 2004; Eckhart et al. 2006; Zhang et al. 2017). Among them, FCC is the most effective and most important regulatory strategy and is often accompanied by the cessation or reduction of floral rewards, because FCC can directly regulate pollinators' foraging behavior using visual signals by serving as a long‐distance attractant and a short‐distance cue for distinguishing floral phases (Weiss 1991, 1995; Oberrath and Böhning‐Gaese 1999; Eckhart et al. 2006).

Usually, the pollen deposition on stigma is jointly affected by two reciprocal pollen transfer processes (pollen receipt and removal) under the two driving forces: improving pollination efficiency and reducing geitonogamy. This phenomenon makes it difficult to distinguish the specific contribution of the two simultaneous processes, leading to most of the adaptive studies of FCC still being speculative based on the visiting behavior of pollinators (Ida and Kudo 2003, 2010; Sun et al. 2005; Nuttman et al. 2006; Makino and Ohashi 2016; Paine et al. 2019; Humphreys and Skema 2023). In *A. guttata*, due to the heteromorphism of pollen‐stigma morphology, the ecological effects of FCC were evaluated by accurately counting intra‐ and inter‐ morph pollen on stigmas for the first time in a natural population. Based on the investigation of the floral morph frequency and heteromorphic incompatible system of the population, the frequency of S‐morph was higher than that of L‐morph, and exhibited weak self‐ and intra‐incompatibility. The role of FCC in reducing geitonogamy is of great significance for maintaining the composition and frequency of floral morphs in the population. Pollinators' excessive visiting can trigger a series of adverse reproductive consequences. Thus, the ability of plants to guide pollinator behavior through FCC is a key adaptive mechanism. Old flowers that do not shed after FCC can not only improve reproductive efficiency by avoiding pollinators' excessive visiting, but also have a certain protective function for pollen tube growth (Delph and Lively 1989; Zhang et al. 2012). For example, in some plants, the adaptive significance of FCC was presumed to reduce the impact of excessive visiting on pollen germination and pollen tube growth (Sun et al. 2005; Zhang et al. 2012). In *A. guttata*, in addition to the above ecological effects, it is not clear whether FCC has any other functions. In the future, we will further explore these questions.

## 5 Conclusion

The present study reports a phenomenon, an inducement and the resulting ecological effects of FCC in a typical distylous species with a heteromorphic pollen‐stigma morphology. The FCC is closely related to the receipt and removal of pollen, which shows that the fading time can be significantly prolonged by limiting the receipt or removal of pollen. Flowers with spots, whether the spots are natural or artificial, show higher attraction to pollinators. However, the excessive visiting of insects did not increase the number of compatible pollen grains, but increased that of incompatible pollen grains. The ecological function of FCC aimed at reducing the self‐pollen deposition on stigmas, that is, geitonogamy.

## Author Contributions

**Dengfu Ren:** data curation (equal), methodology (equal), software (equal), writing – original draft (lead), writing – review and editing (equal). **Rongqian Zheng:** data curation (equal), investigation (equal), methodology (equal), writing – original draft (equal), writing – review and editing (equal). **Yaxin Zhai:** data curation (equal), investigation (equal), methodology (equal). **Aiqin Zhang:** data curation (equal), funding acquisition (lead), investigation (equal), methodology (lead), project administration (lead), resources (lead), software (equal), supervision (lead), validation (lead), writing – original draft (equal), writing – review and editing (lead).

## Funding

This work was supported by the National Natural Science Foundation of China‐regional science foundation project (grant no. 32360308).

## Conflicts of Interest

The authors declare no conflicts of interest.

## Supporting information


**Data S1:** ece372599‐sup‐0001‐DataS1.zip.

## Data Availability

The datasets used and/or analyzed during the current study are available in the supporting information.
